# The effects on self-efficacy, motivation and perceived barriers of an intervention targeting physical activity and sedentary behaviours in office workers: a cluster randomized control trial

**DOI:** 10.1186/s12889-021-11083-2

**Published:** 2021-06-02

**Authors:** Victoria Blom, Emma Drake, Lena V. Kallings, Maria M. Ekblom, Carla F. J. Nooijen

**Affiliations:** 1 The Swedish School of Sport and Health Sciences, Stockholm, Sweden; 2 Division of Insurance medicine, Department of Clinical Neuroscience, Karolinska institutet, Stockholm, Sweden; 3 Department of Psychology, Stockholm university, Stockholm, Sweden

**Keywords:** Self-efficacy, Motivation, Office-workers, Randomized intervention, Physical activity, Sedentary behaviour

## Abstract

**Background:**

The importance of physical activity on health is clear, but changing behaviour is difficult. Successful interventions aiming to improve physical activity and reduce sedentary behaviour is therefore of importance. The aim of this study was to evaluate effects on motivation, self-efficacy and barriers to change behaviour from two different behavioural intervention focusing either on reducing sedentary behaviour or on increasing physical activity as compared to a waiting list control group.

**Methods:**

The study was designed as a cluster randomized control trial (RCT) within two private companies. Self-efficacy, motivation and perceived barriers were together with demographic variables assessed before and after a 6-month intervention. Participant cluster teams were randomly allocated to either the physical activity intervention (iPA), the sedentary behaviour intervention (iSED), or control group. The intervention was multi componential and included motivational counselling based on Cognitive behaviour therapy and Motivational interviewing, group activities and management involvement. Group differences were determined using Bayesian multilevel modelling (parameter estimate; credible interval (CI)), analysing complete cases and those who adhered to the protocol by adhering to at least 3 out of 5 intervention sessions.

**Results:**

After the intervention, the complete cases analysis showed that the iPA group had significantly higher autonomous motivation (0.33, CI: 0.05–0.61) and controlled motivation (0.27, CI: 0.04–0.51) for physical activity compared with the control group. The iSED group scored less autonomous and controlled motivation compared to the iPA group (0.38, CI: − 0.69- -0.087 respectively − 0.32, CI: − 0.57-0.07) but no significant differences compared with the control group. Among individuals that adhered to the protocol, the results showed higher scores on Exercise (3.03, CI: 0.28–6.02) and Sedentary self-efficacy (3.59, CI: 0.35–7.15) for individuals in the iPA group and on Sedentary self-efficacy (4.77, CI: 0.59–9.44) for the iSED group compared to the control group.

**Conclusion:**

These findings indicate that the interventions were successful in increasing self-efficacy in each intervention group and autonomous motivation for exercise in the iPA group, in particular when actively participating in the motivational counselling sessions.

## Background

Physical inactivity and a sedentary lifestyle are related to increased risk of both morbidity and mortality from several non-communicable diseases [1–3]. Furthermore, engaging regularly in physical activity can alleviate symptoms of mental ill-health [4]. Office workers is a group characterised by a sedentary lifestyle at work [5], and an important target group for interventions aiming to improve physical activity and decrease sedentary time.

In terms of designing successful physical activity and sedentary behaviour interventions, is it of importance to understand which mechanisms are involved in reaching behaviour change [6]. Psychological mechanisms that have been shown to be important for behaviour change [6] are to increase self-efficacy [7–9] motivation [10], and limiting perceived barriers for behaviour change [5].

Self-efficacy is defined as “the belief in one’s capabilities to organize and execute the course of action required for producing given attainments” [11]. Exercise Self-efficacy has been shown to be correlated with level of physical activity [12], considered to be the best predictor of physical activity behaviour among employees [13], and shown to mediate the association between intervention and behaviour change in physical activity [8]. It is thus possibly an important mechanism in the process of changing into a more active lifestyle [14] and therefore it is possibly beneficial to attempt to increase the level of self-efficacy in interventions. Factors that increase self-efficacy are for instance feedback on own performance, to take small steps of behaviour change and vicarious experience, i.e. to see other individuals who are similar to you performing the behaviour [9]. To our knowledge self-efficacy towards changing sedentary behaviour has not been studied previously but is supposed to show the same pattern as physical activity as another health behaviour.

The Self-determination theory, and the sub-theory Organismic integration theory [15], suggests that when motivation becomes more self-determined, it facilitates engaging and maintaining behaviours like exercise [10]. The spectrum goes from amotivation, when individuals are not motivated to engage in the behaviour at all, to intrinsic motivation where the individuals participate in the activity for inner incentives such as the joy of doing it. Increasing internalized self-determined motivation is considered important for starting and maintaining behaviours [10]. Furthermore, the theory distinguishes between autonomous motivation and controlled motivation. Controlled forms of motivation are predominantly when activity is perceived primarily as a mean to achieve or getting something and is more characterised of feelings like “having to” compared to “wanting to” [15]. Autonomous motivation includes intrinsic motivation, and refers also to activities perceived inherently fun or satisfying or that give personal value and utility [15]. Earlier findings show consistent support for a positive relation between autonomous forms of motivation and physical activity [16]. Factors that increase autonomous motivation are for instance setting goals in line with emotional values in life [10].

To enable behaviour change, individuals need to overcome their perceived barriers for the activity. Barriers for physical activity may be low self-efficacy and controlled motivation. Some of the most common barriers to breaking up sitting at work has been shown to be sitting is a habit and standing is uncomfortable and tiring [5]. Achieving lower perceived barriers might facilitate behavioural change.

In 2018 and 2019 two different multi-component interventions, incorporating individual, environmental and organizational changes, were performed to increase physical activity or reduce sedentary behaviour among office-workers in order to improve mental health and cognition [17]. The individual intervention comprised of motivational counselling based on Cognitive behaviour therapy including evidence based techniques for behaviour change [6, 18], such as to identify and overcome the individual’s barriers for changing physical activity pattern and setting valued goals to promote autonomous motivation as well as taking small steps of behaviour change to increase individuals’ self efficacy. In a previous study [19], the effectiveness of the intervention was examined in terms of changes in physical activity and sedentary behaviour. The 6-month intervention did not show any significant changes in activity behaviour. However, it is important to investigate the psychological mechanisms in the intervention process.

The aim of the present study is therefore to evaluate if the intervention had an effect on self-efficacy (for physical activity and sedentary behaviour), motivation (for physical activity) and perceived barriers (regarding sedentary behaviour).

## Methods

### Study design

This study is part of a six-month cluster randomized control trial targeting physical activity and sedentary behaviour among office workers to promote healthy brain functions. The intervention consisted of three groups: the physical activity intervention group (iPA), the sedentary behaviour intervention group (iSED) and the control group (C). The intervention was carried out in 2018 and 2019. The detailed study protocol has earlier been published [17]. The trial was prospectively registered as ISRCTN92968402 on 27/02/2018, recruitment started 15/03/2018. Ethical approval was granted by The Stockholm Regional Ethical Review Board (2017/2409-31/1). All participants provided written informed consent before first data collection.

### Participants and data collection

Office workers from two private Swedish companies with around 2000 employees were invited. Inclusion criteria were to be between 18 and 70 years and to have the capability of standing and exercising. Individuals were also excluded from the study at baseline if they showed a very high physical activity level assessed by accelerometer: more than 30 min of moderate to vigorous physical activity per day in prolonged bouts (≥10 min). For further information, please see Nooijen et al. [17]. In the study population the mean age was 42 years and 73% were women. The participants worked within various office professions such as sales, IT, marketing, HR and economy.

The participants were allocated to a cluster group within their company, grouped by human resources personnel at each company. The aim was that a cluster group should share team or line manager, have regular group meetings and limited regular meetings with other groups. The 22 clusters were then randomized into the three different intervention groups: iPA, iSED or control group. The research assistants working with the data collection were blinded for group allocation.

### Intervention and motivational counselling

The two interventions were based on ecological framework targeting multiple intervention levels, including individual, organizational and environmental [20, 21]. The intervention aimed to target both work- and leisure time. On the individual level, the participants received motivational counselling consisting of five counselling sessions based on Cognitive behavioural therapy (CBT) and Motivational interviewing (MI), three individual- and two group sessions. The counselling sessions were performed by health coaches from a health promotion company, who received additional training on CBT techniques and on physical activity and sedentary behaviour by CBT educated psychologists and physical activity experts. The CBT techniques included in the motivational counselling were 1) exploring and setting valued goals of the individual’s own emotional reasons to change physical activity pattern, 2) functional analysis to explore what triggers and maintains the present behaviour and 3) acceptance techniques to deal with potential unpleasant feelings that may come with new behaviours. These are all evidence based CBT techniques chosen to target the individual’s behaviour change of physical activity pattern during their 24 h movement behaviour both at work and private life [18]. The participants also registered their physical activity pattern and received feedback on their activity behaviour [6]. One team leader per cluster were recruited with the aim to encourage the employees to participate, remain in the study and communicate with the research team. Moreover, on environmental level, the participants in the iPA group received access to a commercial gym for 6 months, exercise and lunch walks organized by team leaders as well as access to company bikes to be used for active transport. In the iSED, the team leaders instead initiated standing and walking meetings and encouraged to use sit-stand desks. On the organizational level, team leaders encouraged their employees to be physically active and reduce sedentary behaviour at work and outside work, including commuting to work. The intervention and the motivational counselling is described more fully in the protocol article [17].

### Self-efficacy

Exercise self-efficacy was assessed with a ten item question using the Swedish version of the Exercise Self-efficacy Scale [7]. The scale contains questions regarding the confidence the person feels for performing exercise and physical activity. The response alternatives consist of a four-point scale: 1 = not at all confident; 2 = somewhat confident; 3 = confident; 4 = totally confident. The possible scores ranged from 10 to 40, with a higher value indicating higher self-efficacy. The scale has shown high reliability and internal consistency among adults with neurological disease [7]. However, the reliability and validity has not been tested in a healthy working population. Sedentary Self-Efficacy Scale was an additional scale developed by the authors based on the Exercise Self-efficacy Scale. The reliability and validity of this scale has not been examined, but the scale follows the same structure as the Exercise Self-Efficacy Scale but exchanging the word physical activity to sedentary behaviour. Median values were imputed for missing values for participants with one or two values missing in their Self-Efficacy scores (*n* = 22 in total). The missing values were evenly distributed between individuals and items.

### Motivation to physical activity

The Behavioural Regulation in Exercise Questionnaire [10], BREQ-4 Short version, was used to measure motivation to physical activity. The original response scores range from one to seven, but we used a five-point Likert scale, ranging from 1 = “I don’t agree at all” to 5= “I completely agree” to align to the other items in the questionnaire. BREQ-4 measures behavioural regulations with 7 factors; amotivation, external regulation, introjected avoidance, introjected approach, identified regulation, integrated regulation and intrinsic regulation. A mean score for autonomous motivation was created by combining intrinsic, integrated and identified regulation and for controlled motivation external regulation, introjected avoidance and introjected approach was combined, according to the psychometric properties of the measure [10].

### Perceived barriers to reduce sedentary behaviour

An earlier publication has descriptively examined perceived barriers to decrease sedentary behaviour among Swedish office workers [5]. The study examined 13 different reasons why individuals were sedentary during working hours. Three barriers were found particularly common, “sitting is a habit” (67%), “standing is uncomfortable” (29%) and “standing is tiring” (24%). These three barriers were used in present study. The response alternatives are binary, “yes” or “no”.

### Covariates

Included covariates were age, education (number of years), gender, and company (2 options).

### Statistical analysis

Mean values and standard deviations are presented for both baseline and 6-month follow-up stratified on group allocation for exercise self-efficacy, sedentary self-efficacy, autonomous motivation, controlled motivation and amotivation, together with percentage of individuals responding yes on each of the three perceived barriers. Independent t-tests and chi-square statistics were used to test for differences in baseline characteristics between drop-outs within the three groups.

Comparison between groups were made with multilevel models using Bayesian statistics, similar to what was presented for the main results of the randomized control trial [22, 23]. In brief, Bayesian regression was used to calculate estimates and 95% credible intervals. The credible intervals can be interpreted as probabilities. Other benefits with Bayesian statistics is the calculation of exact parameters estimates without reliance on large sample size, and that it can be applied to a large range of models, like multilevel models that are used in present study [22, 23].

R statistical program language (3.6.1) were used in Rstudio (1.2.5) for the multilevel analyses using the brms (2.11.1), tidyverse (1.3) and tidybayes (1.1) packages. SPSS version 25 were used for descriptive statistics and the drop-out analyses.

### Models

Clustering of data was in all models taken into account using a two-level structure with clusters as the first and participants as the second level. All groups were simultaneously regressed on the post-test value of the outcome and adjusted for the outcome baseline value. Covariates were age, gender, education, and company. We used uninformative prior (student t) for the coefficients. The Gaussian function was used (4 chains, 4 cores and 3000 iterations, 1000 warm-up) for the outcomes self-efficacy and motivation. Parameter estimates and 95% credible intervals were presented. The Bernoulli function was used (4 chains, 4 cores and 3000 iterations, 1000 warm-up) for the analyses on perceived barriers. Parameter estimates and credible intervals were exponentiated to provide posterior odds ratios. For all models, the achieved level of convergence was Rhat values of 1.

For participants with missing or impossible values, education was imputed with the median value. No further imputations were performed. There were no missing values for gender or age.

For each of the different defined outcome variables, separate models were run and repeated on two datasets: A. Complete Cases (CC): All participants with data at baseline and 6-months. B. Per Protocol (PP): Individual was in iPA or iSED group and had attended 3 to 5 counselling sessions, or individual was in C group. For those individuals who did not have information on attendance available, it was assumed that they did not participate in at least 3 sessions.

## Results

### Descriptives

**Fig. 1:**
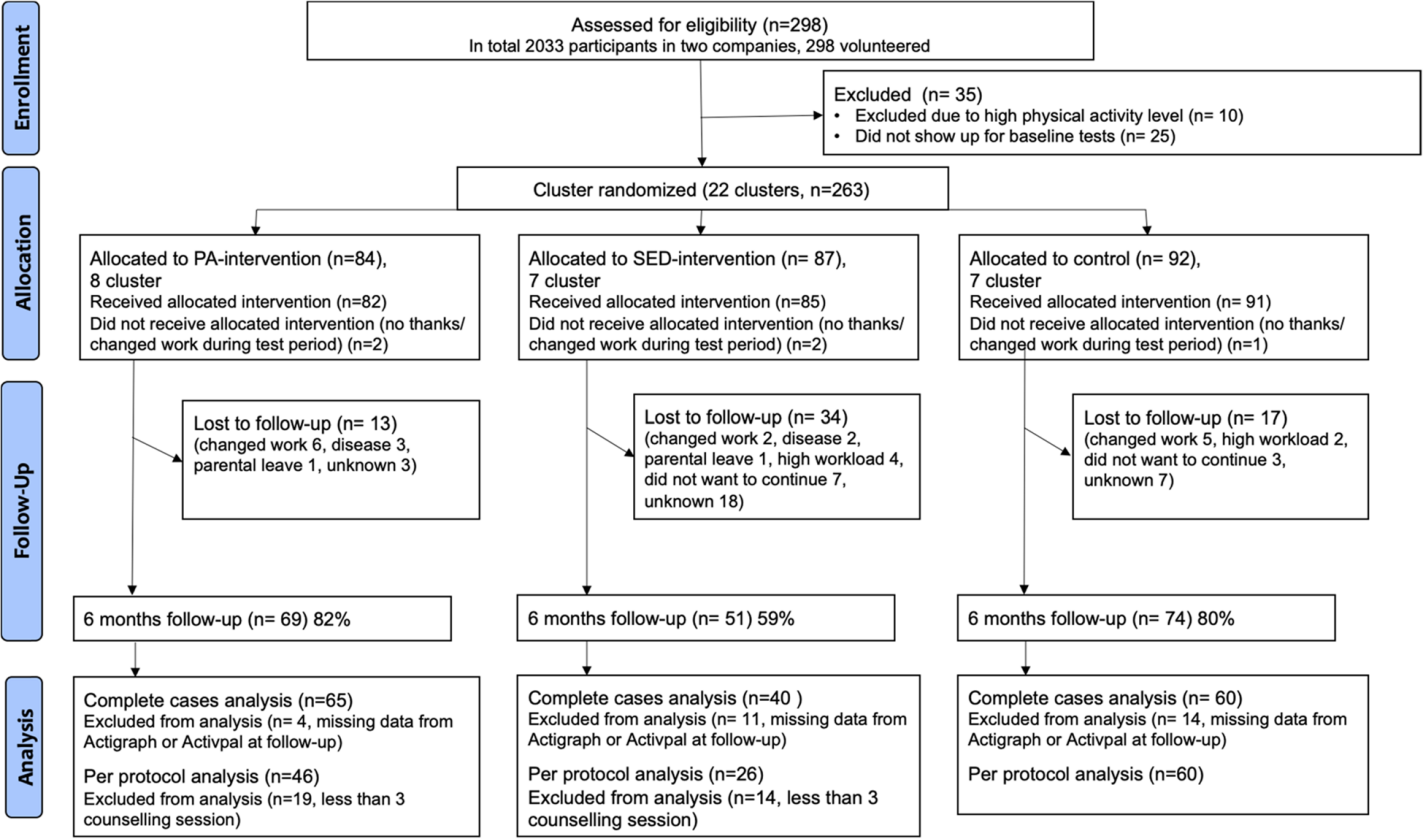
Flowchart illustrating how the analytical sample was reached from the invited individuals. Note: Numbers is for complete cases and per protocol analysis are based on if the participants have data on self-efficacy and motivation

In the CC analysis 27% of participants in the iPA-group, 52% in the iSED-group and 33% in the control group dropped out to follow up (Fig. 1). The distribution of gender, age and education within three groups are presented in Table 1. The MANOVA showed significant differences for age in the complete cases data between the groups. No statistical difference was found between the groups in the per protocol sample.

**Table 1:** Complete cases analysis

	iPA		iSED		C		iPA vs C	iSED vs C	iSED vs iPA
	Baseline mean (SD)	Post-test mean (SD)	Baseline mean (SD)	Post-test mean (SD)	Baseline mean (SD)	Post-test mean (SD)	95% Credible interval		
Self-efficacy^a^	*n* = 60		*n* = 41		*n* = 61				
Exercise Self-efficacy mean, range 10–40	28.0 (7.9)	30.5 (6.9)	28.7 (6.4)	30.1 (7.6)	28.3 (7.5)	28.5 (7.6)	2.00 (−0.497 to 4.55)	0.814 (−1.88 to 3.64)	−1.19 (−3.96 to 1.42)
Sedentary Self-efficacy mean, range 10–40	26.7 (7.6)	27.5 (8.0)	28.0 (6.4)	29.1 (7.6)	26.7 (7.2)	25.4 (7.8)	2.99 (− 0.142 to 6.36)	3.22 (− 0.297 to 6.62)	0.229 (−3.32 to 3.46)
Motivation PA^a^	*n* = 60		*n* = 41		*n* = 61				
Autonomous mean	3.3 (1.2)	3.8 (1.0)	3.4 (1.1)	3.5 (1.2)	3.4 (1.1)	3.5 (1.0)	0.329 (0.052 to 0.606)*	−0.051 (− 0.367 to 0.253)	−0.379 (− 0.690 to − 0.087)*
Controlled mean	2.1 (0.7)	2.4 (0.7)	2.1 (0.6)	2.2 (0.8)	2.0 (0.5)	2.1 (0.7)	0.273 (0.043 to 0.505)*	−0.415 (− 0.297 to 0.213)	−0.315 (− 0.574 to − 0.066)*
Amotivation mean	1.2 (0.1)	1.2 (0.1)	1.4 (0.1)	2.3 (0.1)	1.1 (0.1)	1.2 (0.1)	0.030 (−0.192 to 0.268)	0.026 (−0.216 to 0.283)	− 0.003 (− 0.245 to 0.234)
Barriers SED^b^	*n* = 55		*n* = 40		*n* = 59				
	n (%)		n (%)		n (%)				
Standing is a habit (yes)	47 (85.5%)	43 (78.2%)	32 (80.0%)	30 (75.0%)	51 (86.4%)	49 (83.1%)	2.43 (0.647 to 9.75)	2.30 (0.602 to 9.75)	0.948 (0.242 to 3.59)
Standing is uncomfortable (yes)	26 (47.3%)	20 (36.4%)	14 (35.0%)	9 (22.5%)	24 (40.7%)	25 (42.4%)	1.73 (0.499 to 6.31)	3.45 (0.872 to 15.6)	1.99 (0.508 to 8.70)
Standing is tiring (yes)	24 (43.6%)	11 (20.0%)	15 (37.5%)	8 (20.0%)	21 (35.6%)	17 (28.8%)	3.61 (0.961 to 15.0)	2.62 (0.664 to 11.1)	0.726 (0.170 to 2.88)

*Posterior probability > 0.975 or < 0.025. ^a^ Posterior mean ratio. ^b^ Posterior Odds ratio

Drop-outs between baseline and the 6-month follow-up in the iPA-group (*n* = 22), the iSED-group (*n* = 44) and the control group (*n* = 29) were not different in terms of gender, age, education, baseline exercise self-efficacy or motivation compared to non drop-outs. Drop-outs from the iSED group had significantly lower sedentary self-efficacy at baseline than participants that remained in the study. Individuals actively participating in intervention counselling sessions were not statistically different from the rest of the participants in baseline characteristics, self-efficacy or motivation.

### Multilevel models

Multilevel models on the complete cases data (Table 1) showed that after the intervention, the iPA group had significantly higher autonomous motivation (0.33, CI: 0.05–0.61) and controlled motivation (0.27, CI: 0.04–0.51) for physical activity compared with the control group, and the iSED group scored less autonomous and controlled motivation compared to the iPA group (0.38, CI: − 0.69- -0.087 respectively − 0.32, CI: − 0.57-0.07).

**Fig. 2:**
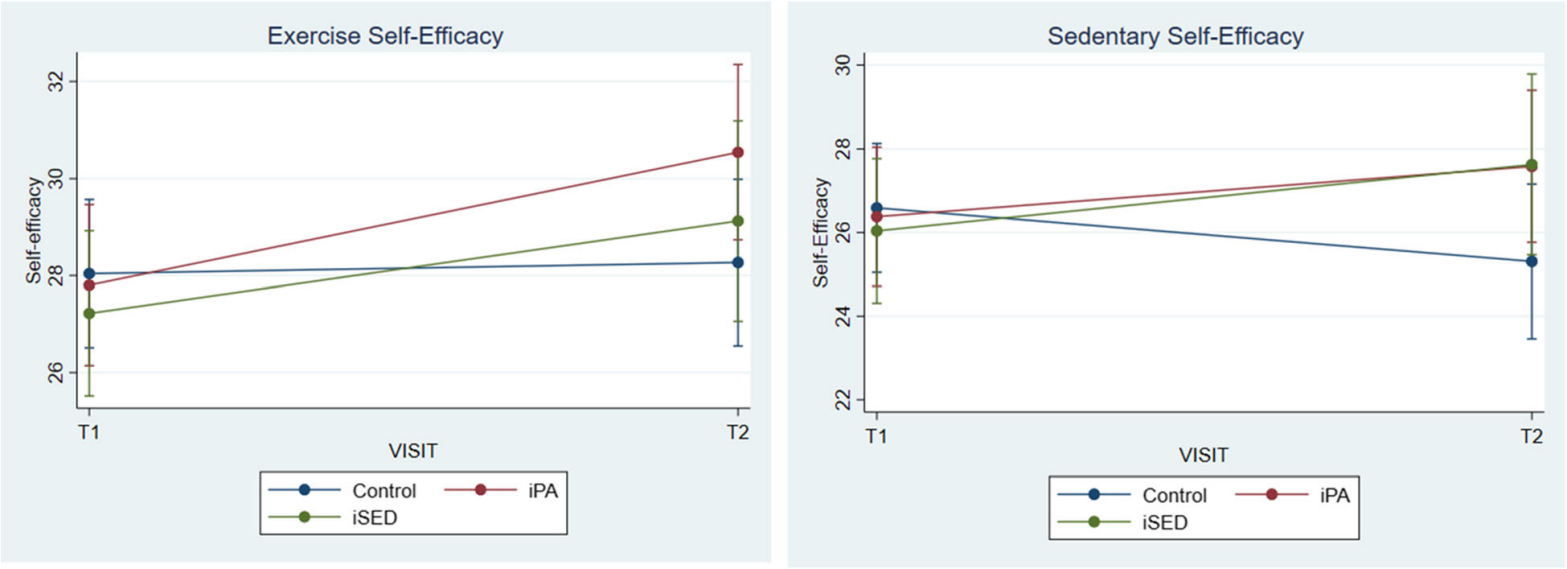
Exercise and Sedentary Self-efficacy pre and post intervention in the iPA, iSED and control group

Among individuals that adhered to the protocol (Table 2), the results showed higher scores on Exercise (3.03, CI: 0.28–6.02) and Sedentary self-efficacy (3.59, CI: 0.35–7.15) for individuals in the iPA group and on Sedentary self-efficacy (4.77, CI: 0.59–9.44) for the iSED group compared to the control group. No group differences were found for controlled motivation, amotivation or perceived barriers (Fig. 2).

**Table 2:** Per protocol analysis

	iPA		iSED		C		iPA vs C	iSED vs C	iSED vs iPA
	Baseline mean (SD)	Post-test mean (SD)	Baseline mean (SD)	Post-test mean (SD)	Baseline mean (SD)	Post-test mean (SD)	95% Credible interval		
Self-efficacy^a^	*n* = 48		*n* = 28		*n* = 61				
Self-efficacy PA	27.3 (8.2)	30.4 (7.1)	29.1 (7.1)	31.2 (6.9)	28.3 (7.5)	28.5 (7.6)	3.19 (0.53 to 5.89)*	2.55 (−0.53 to 5.68)	−0.65 (−3.78 to 2.36)
Self-efficacy SED	26.8 (7.8)	27.4 (7.8)	27.7 (7.2)	29.9 (7.0)	26.7 (7.2)	25.4 (7.8)	3.59 (0.35 to 7.15)*	4.57 (0.77 to 8.60)*	0.98 (−2.93 to 4.74)
Motivation PA^a^	*n* = 44		*n* = 26		*n* = 61				
Autonomous	3.2 (1.2)	3.7 (1.0)	3.4 (1.1)	3.6 (1.1)	3.4 (1.1)	3.5 (1.1)	0.40 (0.11 to 0.69)*	0.10 (− 0.25 to 0.44)	− 0.30 (− 0.64 to 0.03)
Controlled	2.1 (0.7)	2.4 (0.7)	2.0 (0.6)	2.2 (0.9)	2.0 (0.5)	2.1 (0.7)	0.20 (−0.06 to 0.47)	0.02 (− 0.29 to 0.32)	− 0.18 (− 0.49 to 0.12)
Amotivation	1.2 (0.5)	1.1 (0.4)	1.3 (0.9)	1.2 (0.6)	1.1 (0.4)	1.2 (0.5)	−0.08 (− 0.33 to 0.17)	−0.06 (− 0.35 to 0.23)	0.01 (− 0.27 to 0.30)
Barriers SED^b^									
	*n* = 40		*n* = 26		*n* = 59				
Standing is a habit (yes)	32 (80.0%)	31 (77.5%)	22 (84.6%)	19 (73.1%)	51 (86.4%)	49 (83.1%)	2.95 (0.59 to 18.7)	3.41 (0.58 to 22.8)	1.16 (0.19 to 6.37)
Standing is uncomfortable (yes)	20 (50.0%)	16 (40.0%)	8 (30.8%)	8 (30.8%)	24 (40.7%)	25 (42.4%)	1.17 (0.26 to 4.63)	1.58 (0.29 to 8.01)	1.35 (0.26 to 7.33)
Standing is tiring (yes)	16 (40.0%)	9 (22.5%)	10 (38.5%)	6 (23.1%)	21 (35.6%)	17 (28.8%)	4.33 (0.90 to 26.7)	3.18 (0.56 to 20.9)	0.74 (0.13 to 4.00)

*Posterior probability > 0.975 or < 0.025.^a^ Posterior mean ratio. ^b^ Posterior Odds ratio

## Discussion

Changes in self-efficacy, exercise motivation and perceived barriers to reduce sitting time were examined in the present study. Among individuals that participated in at least 3 of 5 counselling sessions, the per protocol results showed higher Exercise self-efficacy for individuals in the physical activity intervention group and a higher score on Sedentary self-efficacy for the sedentary behaviour intervention compared to the control group. Furthermore, individuals in the physical activity intervention showed higher autonomous motivation to exercise after the intervention. The complete cases analyses with all individuals participating in baseline and follow-up measurement (regardless of number of counselling sessions participated in) showed that the physical activity intervention group had higher exercise motivation, both autonomous and controlled, compared with other groups. All together, these findings indicate that the interventions were successful in increasing self-efficacy in each intervention group and autonomous motivation for exercise in the iPA group, in particular when actively participating in the motivational counselling sessions.

To successfully help people change behaviour of physical activity pattern it is important to understand mechanisms for behavioural change in order to design interventions with effective components targeting these mechanisms. In line with previous studies showing that Exercise self-efficacy is important for changes in physical activity patterns [7, 13, 14] self-efficacy was affected by the intervention in the present study. Specifically, it was shown that actively participating in the counselling was important for experiencing higher self-efficacy. The counselling based on CBT and MI involved for instance feedback on physical activity, registration and taking small steps of behaviour change, as social comparison within the cluster teams, all factors that have been shown to be important to change behaviour [6, 9, 18].

Autonomous motivation, i.e. to be motivated by inner incentives, has consistent support for maintaining behaviour change [15] and earlier findings show a positive association between autonomous forms of motivation and physical activity [16]. This is supported by the present study showing that autonomous motivation increased significantly more in the intervention groups compared to the control group. The counselling was especially designed to increase the autonomous intrinsic motivation focusing on individualized support and feedback and setting goals in physical activity patterns that were intimately connected to the individual’s values in life and thus emotionally connected goals promoting autonomous motivation [15]. Moreover, the purpose of the cluster teams were to promote a culture of helping eachother to become more physically active and less sedentary as well as to reinforce the positive behaviour in the group. This may also have contributed to the results of higher autonomous motivation. The Sedentary group showed less autonomous and controlled motivation for exercise compared to the Physical activity group. The logic explanation for this may that the focus in this group was not to increase motivation for physical activity, but to reduce sedentary behaviour.

Achieving lower perceived barriers might facilitate behavioural change. A previous study [5] found that sitting is a habit and standing is uncomfortable and tiring to be the three most common barriers to standing at work. However, even though the intervention was designed to identify and overcome barriers for behaviour change regarding physical activity pattern, the present study did not find any significant between group results regarding changing perception of barriers towards reduced sedentary behaviour.

Even though the present study showed positive changes in self-efficacy and autonomous motivation, a previous study from the research group found that the intervention did not lead to significant changes is physical activity or sedentary behaviour compared to waiting list control group when comparing device-assessed physical activity before the intervention to physical activity after the 6 month intervention [19]. Perhaps this may be explained by the fact that it takes time to change behaviour so that changing self-efficacy and autonomous motivation may be the first changes in the process towards changing behaviour. Future intestigations might also investigate wether factors such as perceived risk of the current behaviour, cognitive functions or work environment might affect the success of behaviour change interventions and/or usefulness of specific components of the intervention.

Strengths of this study include a robust design of a cluster randomized control trial including both a physical activity intervention and the sedentary behaviour intervention, together with a control group. Moreover, individuals were clustered based on their working team which decreases contamination between the intervention groups as they have less interaction with other working teams in terms of meetings even though other social interaction such as lunch may have occured. A study strength is also that the intervention was operated under real-world conditions, which may improve the transferability and applicability of the results to practice.

A limitation that may have affected the results is the higher drop-out rate in the sedentary group which may be due to the participants not voluntarily choosing intervention group. Moreover, the Swedish version of the Exercise Self-Efficacy Scale have shown low absolute reliability among adults with neurological disease [7], which indicates limited ability to measure changes over time. This has not been examinedin a non-clinical sample such as office-workers, but our result should be interpreted in the light of this possible limitation. A limitation is also that a scale for motivation to reduce sedentary behaviour was not included in the study, as such a scale based on Self determination theory is lacking. Some effects were seen among the individuals who participated in at least 3 sessions that was not present in the complete cases. While this might indicate a dose respone in the intervention effect it cannot be excluded that this might also reflect reversed causality. Individuals changing their motivation and self-efficacy to a lesser extent might have been less motivated to participate in the sessions. Even though multiple assessments would have captured potential fluctuations of the mechanisms [24] this was not prioritized in order to reduce burden on participant and risk for higher drop out rates.

Future studies are welcomed to replicate these findings. These may benefit from offering participants possibilities to voluntarily choose intervention group, however, bearing in mind that this also affects the conclusions of RCT effects.

## Conclusion

The importance of physical activity on health is clear, but successfully supporting people in changing their behaviour is difficult. The present study found that this multi-component intervention, including CBT-based counselling support and group- and organizational support for changing behaviours regarding physical activity and sedentary behaviour, was successful in changing individuals’ self-efficacy towards a more health enhancing physical activity pattern. It was also successful in changing autonomous motivation to physical activity, in particular if actively participating in the counselling indicating support for longterm behaviour change. This may have implications on work place interventions having advantages of using CBT-techniques to promote longterm behaviour change regarding physical activity patterns but also other behaviours.

## Data Availability

The datasets generated and/or analysed during the current study are not publicly available due to that the original approval by the regional ethics board and the informed consent from the participants do not include such direct free access, but are available from the corresponding author on reasonable request.
